# Rosavin modulates CX3CL1 secretion and osteoblast morphology under mineralizing conditions in vitro

**DOI:** 10.1007/s11033-026-11980-y

**Published:** 2026-05-22

**Authors:** Piotr Wojdasiewicz, Edyta Wróbel, Elżbieta U. Stolarczyk, Agnieszka Mikulska, Krzysztof Stolarczyk, Dariusz Szukiewicz

**Affiliations:** 1 Department of Biophysics, Physiology and Pathophysiology, Faculty of Health Sciences, Medical University of Warsaw, 5 Chałubińskiego Street, Warsaw, 02-004 Poland; 2 Spectrometric Methods Department, National Medicines Institute, 30/34 Chełmska Street, Warsaw, 00-725 Poland; 3 Faculty of Chemistry, University of Warsaw, 1 Pasteura Street, Warsaw, 02-093 Poland

**Keywords:** rosavin, CX3CL1, fractalkine, osteoblasts, osteoimmunology, bone metabolism

## Abstract

**Background:**

Rosavin, a bioactive compound derived from *Rhodiola rosea*, has been proposed to modulate osteoblast-associated signaling pathways. Emerging evidence highlights the role of CX3CL1 (fractalkine) in bone–immune interactions. This study aimed to investigate whether rosavin modulates CX3CL1 secretion and osteoblast morphology and confluence in human osteoblasts (HOB) in vitro, thereby providing descriptive cellular evidence relevant to CX3CL1-associated osteoblast responses under mineralizing conditions.

**Methods:**

Prior to biological testing, the chemical identity of rosavin was verified by high-resolution electrospray ionization quadrupole time-of-flight mass spectrometry (ESI-QTOF-MS). HOB were cultured in growth medium (DMEM-g), mineralization medium (DMEM-m), or DMEM-m supplemented with rosavin at 50 µM (R50) or 100 µM (R100). Cell morphology and confluence were assessed by phase-contrast microscopy, supported by semi-quantitative image-based estimation of cell-covered area using Fiji/ImageJ. Sirius Red staining was performed only in the DMEM-m mineralization control group and was evaluated qualitatively as a supportive assessment of extracellular matrix maturation.

**Results:**

Rosavin was associated with dose- and time-dependent changes in CX3CL1 secretion and morphology-related observations in HOB cultures. The 50 µM rosavin condition showed significantly increased CX3CL1 secretion at days 14 and 21 compared with DMEM-m alone and was associated with higher cell confluence and predominance of cuboidal cell morphology. In contrast, the 100 µM condition was associated with lower apparent confluence, mixed cellular morphology, and reduced CX3CL1 secretion at later time points.

**Conclusions:**

These observations are descriptive, preliminary, and hypothesis-generating. Rosavin was associated with concentration-dependent modulation of CX3CL1 secretion and osteoblast morphological characteristics in vitro; however, the biological significance of CX3CL1 modulation in this monoculture model remains uncertain. Further mechanistic and functional studies are required.

## Introduction

Bone remodeling depends on coordinated cellular activity and cytokine-mediated communication between skeletal and immune compartments [1, 2]. Chemokines and growth factors shape osteoblast behavior during differentiation and matrix formation, making them relevant readouts in controlled in vitro models of osteogenesis [3, 4].

Among the chemokines relevant to bone metabolism is fractalkine (CX3CL1), a member of the CX3C chemokine family [5]. CX3CL1 is a 37 kDa protein expressed as a membrane-bound form that can be released into the extracellular space through cleavage by metalloproteinases such as a Disintegrin and Metalloproteinase 10 (ADAM10) and ADAM17 [6]. Endothelial cells are its primary source, although monocytes, macrophages, and dendritic cells can also produce it [7]. What makes CX3CL1 unique is its dual mode of action: it functions both as a chemoattractant and as an adhesion molecule. Through binding to its receptor CX3CR1, it mediates leukocyte recruitment and stable adhesion to the endothelium, thereby facilitating diapedesis [8, 9].

Due to its role in leukocyte trafficking, CX3CL1 is generally regarded as a pro-inflammatory chemokine. It has been implicated in the pathogenesis of atherosclerosis, rheumatoid arthritis (RA), and multiple sclerosis [10, 11]. At the same time, it can exert protective effects in the central nervous system by modulating microglial activity and limiting neurotoxicity [12, 13]. Increasing evidence also points to its involvement in bone remodeling and the pathogenesis of osteoporosis [14]. Osteoimmunology highlights chemokines such as CX3CL1 as potential regulators of osteoblast and osteoclast activity [15, 16]. A deeper understanding of its role in osteoblast function remains warranted.

Despite increasing interest in CX3CL1 in bone remodeling and osteoimmune communication, its regulation in primary human osteoblasts remains incompletely characterized. In particular, limited data are available on how bioactive phytochemicals modulate CX3CL1 secretion together with osteoblast morphological characteristics under mineralizing conditions. To date, information regarding the effects of rosavin on human osteoblasts is scarce, and no comprehensive evaluation of CX3CL1 secretion in primary human osteoblasts (HOB) cultures has been reported.

**Fig. 1:**
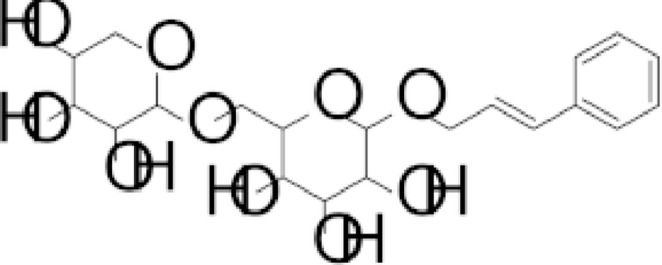
Structural formula of rosavin

In our previous work, we demonstrated that rosavin (Fig. 1), a characteristic constituent of *Rhodiola rosea* L., enhances Bone Morphogenetic Protein 2 (BMP-2) levels in human osteoblast cultures [17], indicating that rosavin can modulate osteoblast-associated readouts in vitro. While the earlier study focused on BMP-2-related osteogenic pathways, the present investigation addresses a distinct biological mechanism, namely the regulation of CX3CL1 secretion, a cytokine of recognized importance in immuno-skeletal interactions. In this way, the current study complements previous findings and further characterizes rosavin-associated osteoblast responses under mineralizing conditions.

The aim of this study was to characterize osteoblast-specific cellular responses to chemically validated rosavin under mineralizing conditions, with particular focus on CX3CL1 secretion and morphology-related changes in HOB cultures.

## Materials and methods

### ESI-QTOF-MS measurements

A high-resolution mass spectrometer, MaXis 4G from Bruker Daltonik (Bremen, Germany), with a time-of-flight (TOF) analyzer, was used to obtain ESI mass spectra. The TOF analyzer was calibrated using a solution of sodium formate in the range of m/z 50–1500. The following settings were used: ESI positive and negative ion mode: dry gas flow rate 4.0 L min^− 1^, dry heater 200 °C, capillary voltage 4500 V and 3000 V for positive and negative ion mode, respectively, plate offset − 500 V, and MS data full scan mode (from m/z 50 to 1000). The QTOF system was controlled by MAXIS software, and data processing DataAnalysis software Compass 1.3 (Bruker Daltonik).

### Cell culture and experimental setup

HOB (PromoCell, C-12720, Heidelberg, Germany) were obtained from the trabecular bone of the hip and knee joints. The cells were expanded in 25 cm² flasks in Basal Medium (PromoCell, C-27001) supplemented with SupplementMix (PromoCell, C-39615) and 1% penicillin–streptomycin (10,000 U/mL and 10 mg/mL; Sigma-Aldrich, P0781). Cultures were maintained at 37 °C in a humidified atmosphere with 5% CO₂, and the medium was exchanged every three days. At confluence, cells were detached using the DetachKit (PromoCell, C-41200), which contains trypsin/EDTA (0.04%/0.03%), neutralization buffer, and HEPES-buffered balanced salt solution (HEPES-BSS). Only cells from the sixth passage were included in experiments. Seeding was performed in 24-well plates at a density of 4.4 × 10⁴ cells per well, corresponding to a growth area of 1.9 cm², and cultures were allowed to expand until 70–80% confluence. After reaching the required density, cultures were divided into four experimental groups. The control group (DMEM-g) was maintained in growth medium composed of Basal Medium and SupplementMix, whereas the mineralization group (DMEM-m) contained cells cultured in mineralization medium (PromoCell, C-27020) supplemented with its specific SupplementMix (C-39616). To evaluate the influence of rosavin, two further groups were prepared by supplementing the mineralization medium with either 50 µM rosavin (R50) or 100 µM rosavin (R100). Cultures were maintained for 21 days, with medium renewed every three days.

### Rosavin preparation and cytokine measurement

Rosavin (Sigma-Aldrich, SML0336) was supplied as crystalline powder and dissolved in sterile phosphate-buffered saline (PBS; ThermoFisher, 14190169) to generate a 10 mM stock solution. Working dilutions were freshly prepared in mineralization medium immediately before supplementation. Supernatants were collected 72 h after rosavin exposure at defined experimental time points (day 0, 14, and 21). Samples were stored at − 20 °C until analysis. Cytokine concentration was determined using a commercial ELISA kit (Abcam, ab192145, Waltham, MA, USA) according to the manufacturer’s instructions. Fifty microliters of each standard or sample were added to the wells, followed by 50 µL of antibody cocktail, and incubated for 1 h at room temperature. Wells were washed three times with 350 µL of 1× Wash Buffer. Subsequently, 100 µL of 3,3’,5,5’-tetramethylbenzidine (TMB) substrate was applied and incubated for 10 min before stopping the reaction with 100 µL of stop solution. Absorbance was measured at 450 nm using a microplate spectrophotometer (ASYS UVM 340, Biocompare, San Francisco, CA, USA). Control groups received an equivalent volume of PBS corresponding to rosavin-treated conditions.

### Evaluation of collagen deposition, cell confluence, and morphology

To investigate extracellular matrix formation and changes in osteoblast confluence and morphology, collagen deposition and culture morphology were assessed on days 14 and 21. Cells from the DMEM-m group were rinsed three times with calcium–magnesium PBS (ThermoFisher, 14040174) and subsequently incubated with 0.1% Sirius Red solution (Sigma-Aldrich, 365548) for 60 min at room temperature. After staining, plates were washed with 10 mM HCl to remove unbound dye. Sirius Red staining was performed exclusively in the DMEM-m group to qualitatively validate extracellular matrix maturation under mineralizing conditions and was not intended to assess rosavin-specific effects. Therefore, no threshold-based quantitative comparison of collagen deposition between rosavin-treated and untreated cultures was performed.

Phase-contrast microscopy was used to compare cell morphology and surface coverage between the experimental groups. Images were acquired using an inverted phase-contrast microscope (Zeiss Primovert, Jena, Germany) equipped with a Zeiss Axiocam ERc 5s camera. For comparisons within each time point, images were obtained under identical microscope and camera settings, including magnification, illumination, and exposure conditions. Representative non-overlapping fields were selected from each experimental condition.

Supportive semi-quantitative confluence estimation was performed using Fiji/ImageJ software. Images were converted to 8-bit grayscale, and the cell-covered area was segmented using the same thresholding procedure within each experimental series. The threshold was selected to include cell bodies and exclude background areas corresponding to the uncovered culture surface. Binary masks were visually checked against the original phase-contrast images to ensure appropriate segmentation. Confluence was expressed as the percentage of cell-covered area relative to the total image area. This analysis was used only as supportive information for the descriptive assessment of culture confluence and morphology. The confluence estimation was performed in a non-blinded manner. This methodological limitation is acknowledged in the Discussion.

Cell morphology was assessed descriptively, with attention to the predominance of cuboidal or elongated fibroblast-like cells and the presence of dense cell clusters. No predefined morphological grading scale was applied. The assessment was performed in a descriptive manner and should be interpreted as supportive observational evidence rather than as a definitive quantitative morphological endpoint.

### Statistical analysis and ethical consideration

All experiments were performed in three independent biological replicates (*n* = 3), and this number was consistent across all analyzed groups and time points. A biological replicate was defined as an independent culture experiment performed under the same experimental conditions using commercially obtained HOB. The Shapiro–Wilk test was used to assess normality of distribution, and variance homogeneity was evaluated using the Brown–Forsythe test. As the data did not consistently meet parametric assumptions, differences between groups were analyzed using the non-parametric Kruskal–Wallis test. Post-hoc comparisons were performed using Dunn’s multiple comparison test. Statistical analyses were performed using GraphPad Prism (GraphPad Software, San Diego, CA, USA). A p-value < 0.05 was considered statistically significant. The study was conducted exclusively on commercially available HOB. In line with the Declaration of Helsinki [18] and International Society for Stem Cell Research (ISSCR) recommendations [19], no ethics approval was required, as no material was collected directly from human donors and no experiments involved human subjects. The study design did not assess donor-to-donor variability and was not intended to compare cells derived from different donors.

## Results

### Confirmation of the identity of rosavin

**Fig. 2:**
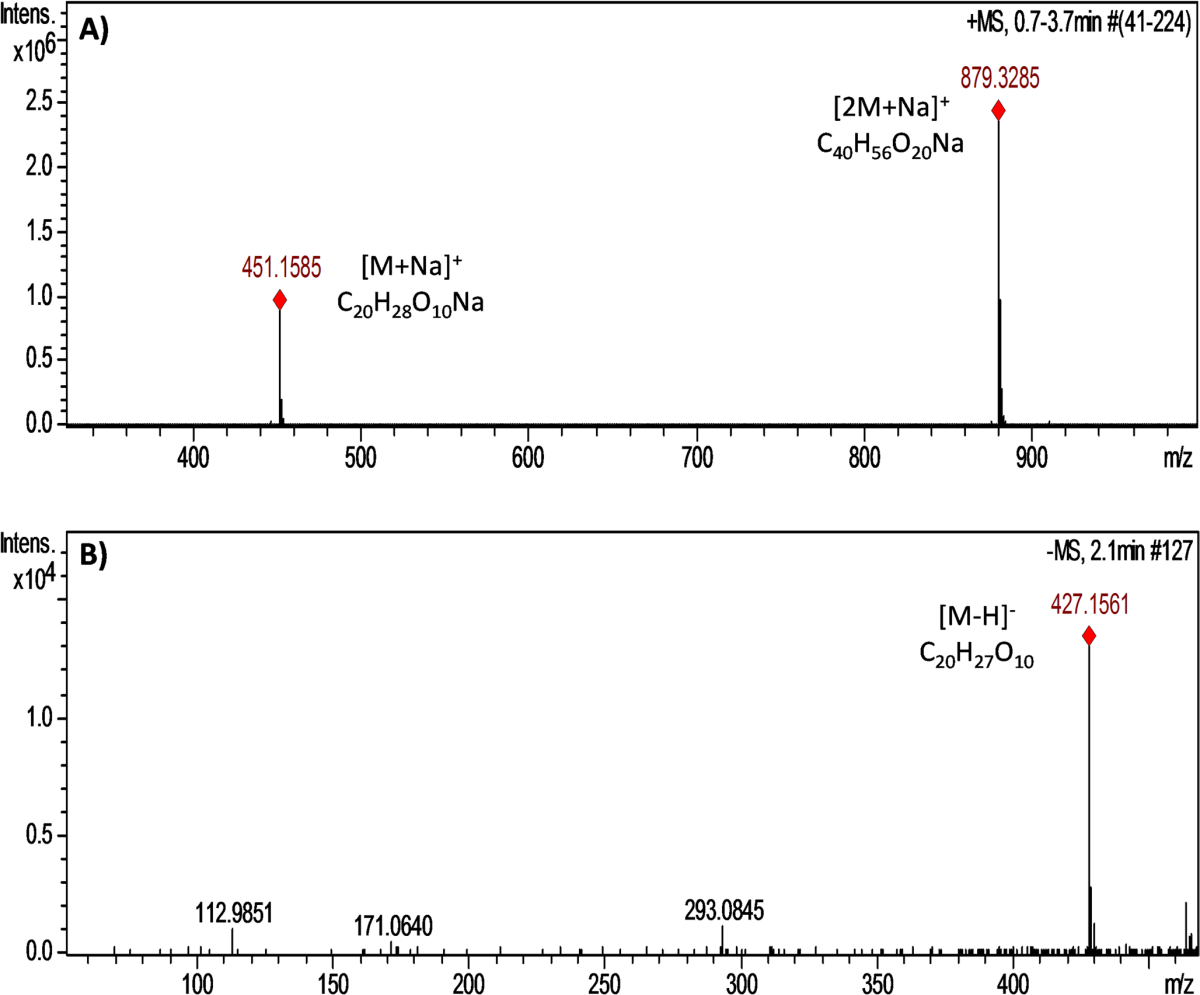
High-resolution mass spectrometry MS spectra of rosavin in (A) positive ionization mode (+ νe); (B) negative ionization mode (- νe)

The purchased rosavin sample was subjected to identity analysis by mass spectrometry (MS) before further studies. Identification of rosavin was based on accurate mass, isotopic distribution, and fragmentation pattern. Figure 2 shows representative ion chromatograms (MS spectra) of the tested sample recorded in positive and negative polarity.

The (+) νe electrospray ionization (ESI) spectrum of the rosavin showed peaks at m/z 451.1585 and 879.3285, for sodium compounds [M + Na]^+^, and sodium adducts [2 M + Na]^+^, corresponding to the molecular formulas C_20_H_28_O_10_Na and C_40_H_56_O_20_Na, respectively. The (-) νe ESI spectrum of the rosavin showed deprotonated molecular ion peaks at m/z 427.1561, corresponding to the molecular formula C_20_H_27_O_10_. The MS results confirmed the molecular identity of rosavin and supported its suitability for subsequent biological experiments.

### CX3CL1 secretion in human osteoblast cultures

**Fig. 3:**
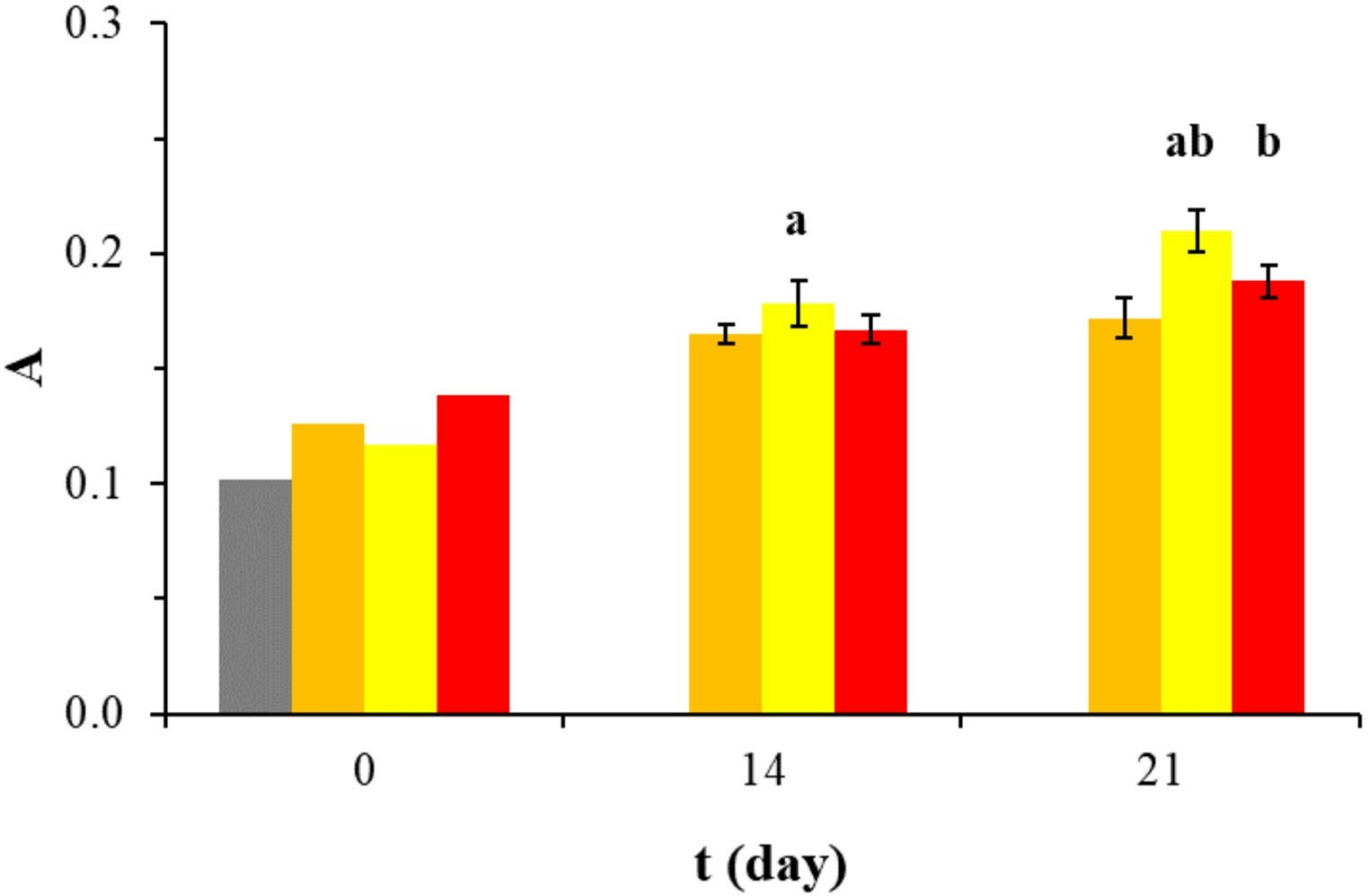
A graph illustrates the changes in mean CX3CL1 secretion, quantified by ELISA, throughout the experiment at different time points (0, 14, and 21 days), depending on the applied cell culture conditions and rosavin supplementation. The groups are color-coded as follows: () gray represents DMEM-g, () orange represents DMEM-m, () yellow represents R50, () red represents R100. Data are presented as mean ± standard deviation (SD), based on three independent biological replicates (*n* = 3). Symbol a indicates a statistically significant difference (*p* < 0.05) between the R50 group and the corresponding DMEM-m control at day 14 and day 21. Symbol b indicates a statistically significant difference (*p* < 0.05) between the R50 and R100 groups at day 21, as determined by Dunn’s post-hoc test following Kruskal–Wallis analysis

A graphical representation of the changes in CX3CL1 levels in HOB in all groups during the experiment is presented in Fig. 3. The figure illustrates the differences in CX3CL1 secretion among DMEM-g, DMEM-m, and R50 or R100 on selected experimental days. At baseline (day 0), CX3CL1 levels were slightly higher in the DMEM-m group compared with DMEM-g. In the mineralization control, CX3CL1 levels increased progressively between days 0, 14, and 21. As the study progressed, an increase in CX3CL1 secretion was observed in the R50 group compared with the other cell cultures. Importantly, treatment with 50 µM rosavin increased CX3CL1 levels on days 14 and 21 compared with the mineralization control (DMEM-m on days 14 and 21). When comparing the 14th and 21st day of exposure in the R50 group to the corresponding DMEM-m controls, an 8% increase in CX3CL1 levels was observed on day 14 and a 22% increase on day 21. When CX3CL1 secretion was compared between the R50 and R100 groups, no significant difference was observed on day 14, whereas a significant difference was detected on day 21. On day 21, the R100 condition was associated with an 11% lower CX3CL1 level compared with the R50 condition. Overall, CX3CL1 levels were higher in the R50 condition at later time points, whereas R100 was associated with lower CX3CL1 levels at day 21.

### Cell confluence and morphological alterations

Phase-contrast microscopy, supported by semi-quantitative estimation of cell-covered area, indicated differences in confluence and morphology between rosavin-supplemented cultures and control groups. By day 14, cultures treated with 50 µM rosavin showed higher apparent surface coverage compared with DMEM-g and DMEM-m controls. This trend persisted until day 21, when R50 cultures nearly achieved complete monolayer confluence. In contrast, cultures exposed to 100 µM rosavin showed a less uniform pattern, with lower apparent surface coverage than the R50 group.

**Fig. 4:**
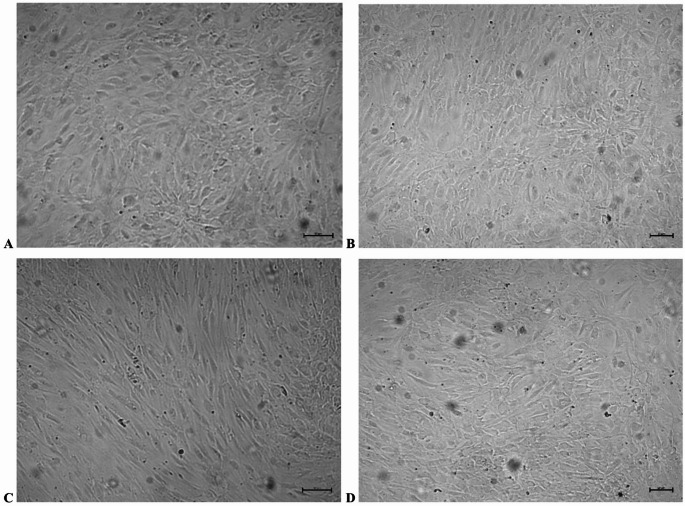
Morphological alterations and confluence of HOB cells during in vitro differentiation in the presence of rosavin (50 µM or 100 µM). Phase-contrast microscopy was applied on day 14 (A, C) and day 21 (B, D) of culture. At 50 µM rosavin, HOB cells showed predominance of cuboidal morphology and high apparent confluence by day 14 (A). Comparable morphological characteristics were maintained on day 21 (B). In contrast, the 100 µM condition was associated with predominantly elongated, fibroblast-like morphology on day 14, with focal regions of densely packed cuboidal cells (C). By day 21, this mixed cellular morphology persisted, characterized by fibroblast-like cells interspersed with high-density clusters of cuboidal cells (D). The apparent confluence at 100 µM was lower than that observed in cultures maintained with 50 µM rosavin. The presented images are representative of independent experimental replicates. Scale bars: 50 μm (day 14); 20 μm (day 21)

**Fig. 5:**
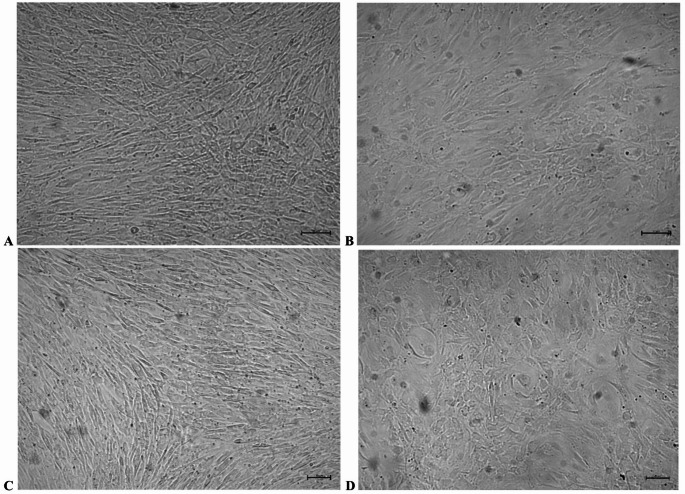
Morphological characteristics of HOB cells during growth and differentiation in vitro. Cells were maintained either in growth medium (A, C) or in mineralization medium (B, D). Phase-contrast images were collected on day 14 (A, B) and day 21 (C, D) of culture. In growth medium, HOB cells predominantly displayed a fibroblast-like morphology, whereas cultures exposed to mineralization medium acquired a more cuboidal morphology typical of cells maintained under mineralizing conditions. A progressive increase in confluence was evident at day 21 compared to day 14. The images shown are representative of independent experimental replicates. Scale bars: 50 μm (day 14); 20 μm (day 21)

Morphologically, R50 cultures were characterized by cuboidal cells forming dense, rosette-like structures. The R100 group showed mixed cellular morphology, with both cuboidal and elongated fibroblast-like cells. In control groups, elongated cells predominated throughout the observation period, and confluence was delayed until day 21. These differences are illustrated in Figs. 4 and 5.

### Collagen deposition during osteoblast differentiation

**Fig. 6:**
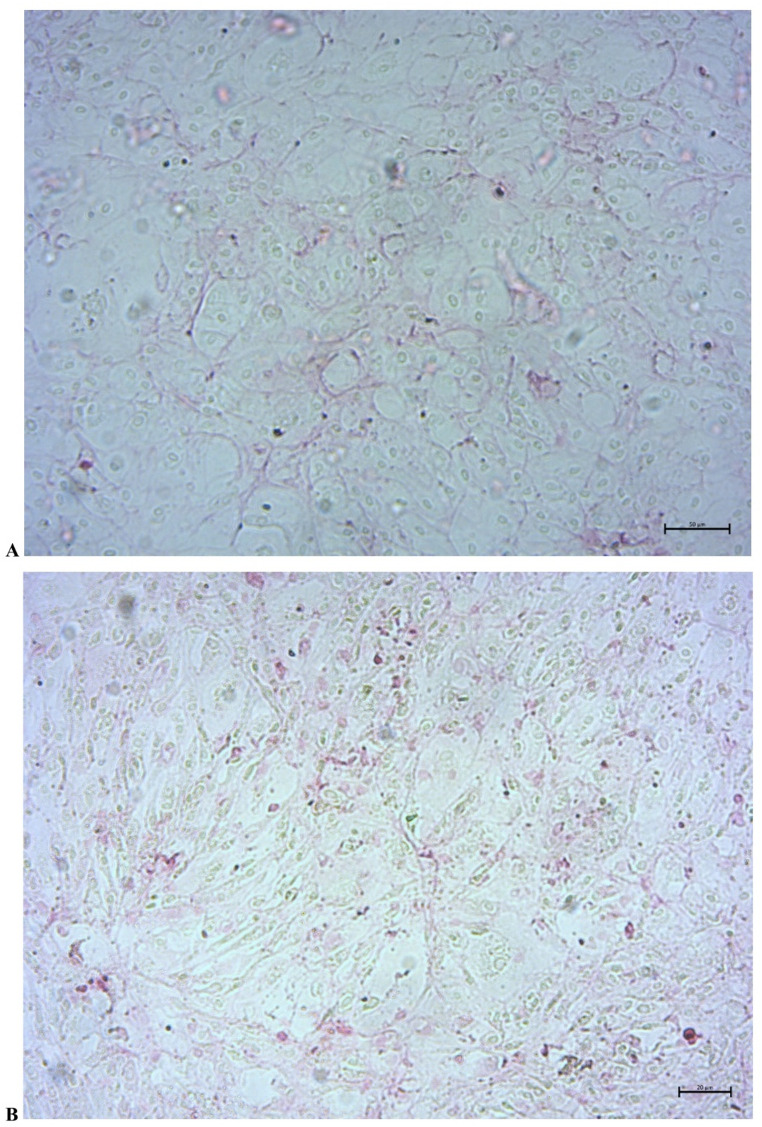
Collagen localization during HOB differentiation on days 14 (A) and 21 (B). Collagen deposition, visualized by purple staining, was evident in the extracellular matrix, serving as a structural framework essential for subsequent mineralization. The images represent findings from independent experiments. Scale bars: 50 μm (day 14); 20 μm (day 21)

Analysis of collagen distribution during osteoblast differentiation revealed progressive extracellular matrix deposition over time. On day 14, purple staining indicated the initial presence of collagen fibers within the extracellular space, forming a supportive scaffold around HOB maintained in DMEM-m. By day 21, the intensity and distribution of staining markedly increased, suggesting more pronounced collagen staining and matrix organization. This progressive enhancement in collagen deposition corresponds to the maturation phase of HOB and provides a structural framework that facilitates subsequent mineralization processes. These qualitative observations indicate progressive extracellular collagen staining during the differentiation timeline under mineralizing conditions (Fig. 6). This pattern confirms the expected maturation of extracellular matrix formation and supports the suitability of the applied in vitro model for longitudinal assessment of osteoblast matrix development. Sirius Red staining was used as a qualitative validation of matrix maturation and was not intended to assess rosavin-specific effects. Because Sirius Red staining was performed only in the DMEM-m group and was not quantified by dye extraction or threshold-based densitometry, these findings should be interpreted as qualitative confirmation of matrix maturation rather than as a comparative analysis of rosavin-specific effects on collagen deposition.

## Discussion

The present study indicates that rosavin is associated with concentration- and time-dependent modulation of CX3CL1 secretion and morphology-related features in HOB cultures maintained under mineralizing conditions. Supplementation with 50 µM rosavin increased CX3CL1 secretion compared with the corresponding mineralization medium control at later time points, with the effect becoming more pronounced between day 14 and day 21. This condition was also associated with higher apparent confluence and predominance of cuboidal cell morphology. In contrast, cultures exposed to 100 µM rosavin showed a less uniform cellular pattern, lower apparent confluence, and reduced CX3CL1 secretion at day 21 compared with the 50 µM condition. These findings should be interpreted as descriptive and hypothesis-generating rather than as evidence of a confirmed osteogenic or mechanistic effect.

Before the biological assays, the identity of rosavin was verified by high-resolution electrospray ionization quadrupole time-of-flight mass spectrometry (ESI-QTOF-MS). This analytical step was essential to confirm the molecular integrity of the compound and to ensure that the observed cellular responses could be attributed solely to rosavin itself rather than to potential contaminants or degradation products. Such chemical validation strengthens the reliability of subsequent in vitro observations and supports the reproducibility of the experimental model.

Previous studies have mainly focused on the osteogenic potential of rosavin through the regulation of growth factors, including the upregulation of BMP-2 in HOB [17]. The current findings expand this perspective by showing that CX3CL1 secretion is modulated in osteoblast cultures exposed to rosavin under mineralizing conditions. CX3CL1, primarily known as a chemokine mediating leukocyte adhesion and migration, has been increasingly implicated in bone resorption and remodeling processes [20]. Our data show that rosavin is associated with increased CX3CL1 secretion in osteoblast cultures under mineralizing conditions, providing descriptive evidence that rosavin can modulate osteoblast-derived immuno-skeletal signaling. These findings indicate that rosavin modulates multiple osteoblast-associated readouts in this in vitro model. Importantly, increased CX3CL1 secretion observed in this study should not be interpreted as inherently osteoanabolic or beneficial, as CX3CL1 has been reported to exert both pro-resorptive and regulatory effects in bone biology. The magnitude of CX3CL1 changes observed in this study was moderate and should be interpreted within the context of an in vitro model. Importantly, the present study does not demonstrate osteogenic enhancement. The analyzed endpoints were limited to CX3CL1 secretion and qualitative observations of morphology and confluence, whereas no functional osteogenic assays, gene expression analyses, or mechanistic pathway studies were performed. Therefore, the biological relevance of the observed CX3CL1 modulation remains uncertain and should not be interpreted as evidence of improved osteogenesis. Rather, the present findings should be regarded as descriptive, preliminary, and hypothesis-generating within a simplified osteoblast monoculture model.

It should be emphasized, however, that many reports describe a catabolic role of CX3CL1 within the skeletal system. As a pro-inflammatory cytokine, CX3CL1 can act in a pro-osteoporotic manner by stimulating osteoclasts and indirectly suppressing osteoblast activity [14, 21]. Within the context of the present model, in which only osteoblasts were studied, it remains difficult to determine the functional implications of rosavin-induced CX3CL1 increase under physiological conditions that also involve osteoclasts. It is important to note, nevertheless, that such a simplified, linear view of CX3CL1 function may be misleading. Its chemotactic properties are also exploited by other cell types—for example, tumor cells, thereby promoting metastasis [22, 23], or glial cells, where CX3CL1 supports repair processes, particularly in neural tissue [24–26]. The functional consequences of osteoblast-derived CX3CL1 modulation were not addressed in this monoculture model and require dedicated assays and/or co-culture systems in future studies.

Another critical aspect of our study is the non-linear nature of the response. Whereas the 50 µM concentration was associated with higher confluence and a more pronounced cuboidal morphology, the higher dose (100 µM) produced less favorable effects, including reduced confluence and heterogeneous morphology. Such non-linear responses have been described for many natural compounds [27–29]; however, in the present study, interpretation of this pattern as a hormetic-like response remains tentative in the absence of direct viability and mechanistic data. This highlights the importance of dose optimization in future in vitro and mechanistic studies of rosavin-exposed osteoblast cultures. Importantly, the reduced response observed at 100 µM rosavin should not be interpreted as evidence of cytotoxicity alone. Two non-mutually exclusive explanations should be considered. First, the lower confluence and reduced CX3CL1 secretion may reflect decreased cell viability, impaired proliferation, or stress-related effects at the higher concentration. Second, they may also reflect an altered differentiation state or a shift in osteoblast phenotype, as suggested by the heterogeneous morphology observed in the R100 group. The present experimental design does not allow these possibilities to be distinguished. Therefore, the 100 µM condition is described here as producing a less favorable cellular profile rather than as a definitively cytotoxic concentration. Future studies should include direct viability and cytotoxicity assays, proliferation markers, and osteogenic differentiation endpoints to clarify this issue. Morphological observations further support this conclusion. In the R50 group, cuboidal cells predominated, forming dense rosette-like arrangements composed mainly of closely apposed cells. In contrast, R100 cultures contained clusters of cuboidal cells interspersed with elongated, fibroblast-like elements, suggesting a less uniform morphological pattern than that observed in the R50 group. Control groups without rosavin did not achieve comparable density or morphology under the present experimental conditions, supporting the view that rosavin exposure was associated with distinct cellular observations in this model. Notably, these results are consistent with previous reports, cautiously supporting the reproducibility of rosavin’s influence on HOB colonies, which we consider an added value of this work and an encouragement for further studies [17].

Parallel Sirius Red staining in the DMEM-m control confirmed the expected increase in extracellular collagen between day 14 and day 21, consistent with matrix maturation under mineralizing conditions described in the literature [30]. This supports the suitability of the applied culture conditions for longitudinal observation of osteoblast matrix development and provides context for interpreting the accompanying changes in CX3CL1 levels, morphology, and confluence.

The observed association between rosavin exposure, CX3CL1 secretion, and changes in confluence and morphology suggests that this compound may influence osteoblast-associated cellular responses at the interface of immunology and bone biology. CX3CL1 is increasingly recognized as a mediator of osteoblast–osteoclast communication and coordinated bone remodeling [14, 16, 31]. In the present monoculture model, rosavin-induced modulation of CX3CL1 secretion reflects altered osteoblast-derived signaling; however, direct effects on osteoclast recruitment or activity were not assessed. Such findings indicate a concentration-dependent osteoblast response that warrants further mechanistic investigation into downstream signaling pathways and intercellular cross-talk. Extrapolation to systemic bone remodeling processes requires further investigation in more complex experimental systems.

Several limitations should be acknowledged. The experiments were performed in vitro using commercially obtained HOB cultures, which do not reproduce the complexity of the in vivo bone microenvironment involving osteoclasts, immune cells, vascular components, and systemic regulatory factors. The study focused primarily on CX3CL1 secretion and supportive morphology-related observations, without mechanistic pathway analysis, gene expression profiling, functional osteogenic assays, or direct viability/cytotoxicity testing. The image-based confluence assessment was supportive, semi-quantitative, and non-blinded, whereas morphology was evaluated descriptively without a predefined grading scale. Sirius Red staining was performed only in the DMEM-m control group as a qualitative validation of matrix maturation and was not designed to assess rosavin-specific effects on collagen deposition. These limitations restrict the biological interpretation of the findings and preclude conclusions regarding osteogenic enhancement, cytotoxicity, or extracellular matrix modulation by rosavin. Accordingly, the present results should be regarded as preliminary and hypothesis-generating.

In conclusion, rosavin was associated with concentration-dependent modulation of CX3CL1 secretion and morphology-related characteristics in HOB cultures maintained under mineralizing conditions in vitro. The 50 µM condition was associated with increased CX3CL1 secretion and a more uniform cuboidal morphology, whereas the 100 µM condition showed a less favorable cellular profile. Because the study did not include functional osteogenic assays, direct viability/cytotoxicity testing, or mechanistic analyses, these results should be interpreted with caution. The findings provide preliminary and hypothesis-generating evidence that may support future mechanistic studies.

## Conclusions

Rosavin was associated with concentration-dependent modulation of CX3CL1 secretion and morphology-related characteristics in HOB cultures maintained under mineralizing conditions in vitro. The 50 µM condition was associated with increased CX3CL1 secretion and a more uniform cuboidal morphology, whereas the 100 µM condition showed a less favorable cellular profile. These observations remain descriptive, preliminary, and hypothesis-generating. Because the study did not include functional osteogenic assays, direct viability/cytotoxicity testing, or mechanistic analyses, the biological significance of the observed CX3CL1 modulation remains uncertain. Further mechanistic and functional studies are required to determine the relevance of these findings in more complex experimental systems.

## Data Availability

The datasets generated and analyzed during the current study are available from the corresponding author on reasonable request.
